# Differentiation syndrome and coagulation disorder — comparison between treatment with oral and intravenous arsenics in pediatric acute promyelocytic leukemia

**DOI:** 10.1007/s00277-023-05270-x

**Published:** 2023-05-18

**Authors:** Jie-Si Luo, Xiao-Li Zhang, Dan-Ping Huang, Yi-Qiao Chen, Wu-Qing Wan, Hui-Rong Mai, Hui-Qin Chen, Hong Wen, Ri-Yang Liu, Guo-Hua Chen, Yu Li, Xue-Qun Luo, Yan-Lai Tang, Li-Bin Huang

**Affiliations:** 1 Department of Pediatrics, The First Affiliated Hospital, Sun Yat-sen University, Guangzhou, Guangdong China; 2 Department of Pediatric Hematology, Fujian Medical University Union Hospital, Fuzhou, Fujian China; 3 Department of Pediatrics, Second Xiangya Hospital, Changsha, Hunan China; 4 Department of Hematology and Oncology, Shenzhen Children’s Hospital, Shenzhen, China; 5 Department of Pediatrics, The Third Affiliated Hospital, Sun Yat-sen University, Guangzhou, China; 6 Department of Pediatrics, The First Affiliated Hospital of Xiamen University, Xiamen, Fujian China; 7 Department of Pediatrics, Huizhou Central People’s Hospital, Huizhou, Guangdong China; 8 Department of Pediatrics, First People’s Hospital of Huizhou, Huizhou, Guangdong China

**Keywords:** Acute promyelocytic leukemia, Children, Arsenic compounds, Differentiation syndrome, Coagulation disorder

## Abstract

Realgar-Indigo naturalis formula (RIF), with A_4_S_4_ as a major ingredient, is an oral arsenic used in China to treat pediatric acute promyelocytic leukemia (APL). The efficacy of RIF is similar to that of arsenic trioxide (ATO). However, the effects of these two arsenicals on differentiation syndrome (DS) and coagulation disorders, the two main life-threatening events in children with APL, remain unclear. We retrospectively analyzed 68 consecutive children with APL from South China Children Leukemia Group-APL (SCCLG-APL) study. Patients received all-trans retinoic acid (ATRA) on day 1 of induction therapy. ATO 0.16 mg/kg day or RIF 135 mg/kg·day was administrated on day 5, while mitoxantrone was administered on day 3 (non-high-risk) or days 2–4 (high-risk). The incidences of DS were 3.0% and 5.7% in ATO (*n* = 33) and RIF (*n* = 35) arms (*p* = 0.590), and 10.3% and 0% in patients with and without differentiation-related hyperleukocytosis (*p* = 0.04), respectively. Moreover, in patients with differentiation-related hyperleukocytosis, the incidence of DS was not significantly different between ATO and RIF arms. The dynamic changes of leukocyte count between arms were not statistically different. However, patients with leukocyte count > 2.61 × 10^9^/L or percentage of promyelocytes in peripheral blood > 26.5% tended to develop hyperleukocytosis. The improvement of coagulation indexes in ATO and RIF arms was similar, with fibrinogen and prothrombin time having the quickest recovery rate. This study showed that the incidence of DS and recovery of coagulopathy are similar when treating pediatric APL with RIF or ATO.

**Supplementary Information:**

The online version contains supplementary material available at 10.1007/s00277-023-05270-x.

## Introduction

With contemporary treatment that combines arsenic compounds, all-trans retinoic acid (ATRA), and anthracycline-based chemotherapy (CHT) [1–4], the long-term survival rate for acute promyelocytic Leukemia (APL) exceeds 90%. However, early death (ED) is still a major issue in APL, due to the two main causes of hemorrhage and differentiation syndrome (DS). In the clinical trial setting, the rate of early hemorrhagic death is about 5–10% [5], while up to 19% of patients develop DS leading to a death rate of up to 5.7% in all patients [5]. Therefore, reducing the incidences of DS and hemorrhage is critical for further increasing the survival rate in patients with APL.

In China, there are two arsenic compounds available for APL: arsenic trioxide (ATO) and Realgar-Indigo naturalis formula (RIF). RIF is a traditional Chinese medicine that contains realgar (As_4_S_4_), Indigo naturalis, Radix *Salviae miltiorrhizae*, and Radix Pseudostellariae which have synergic anti-leukemia effects [6]. The interim analysis of SCCLG-APL study showed that after a median 3-year follow-up, the estimated 5-year EFS was 100%, and adverse events were mild in both RIF and ATO arms. Moreover, RIF is an oral drug that can reduce hospital stay, which is an added advantage. These results were also observed in adult counterparts [3, 4].

Although RIF and ATO have similar effects on the recovery of coagulopathy, their impacts on the early complications may differ in adult patients [7]. Patients treated with RIF have a higher peak white blood cell count (WBC) compared to those treated with ATO during induction treatment [8]. Leukocytosis is an important factor in the development of DS, and there are important distinctions between pediatric and adult patients with APL [9]. In fact, 84–100% of pediatric patients with non-high risk (NHR, diagnostic WBC ≤ 10 × 10^9^/L) APL on ATO and ATRA induction therapy developed leukocytosis (WBC > 10 × 10^9^/L), and this phenomenon is much more common compared to adult counterpart (35–47%) on the same therapy [10–15]. To our knowledge, there is no report comparing the impacts of RIF and ATO on DS and hemorrhage in pediatric patients with APL. Therefore, this study aims to analyze these two main life-threatening events in pediatric patients with APL who received induction therapy with SCCLG-APL (South China Children Leukemia Group-APL) protocol containing ATO or RIF, ATRA, and low-intense chemotherapy [3].

## Materials and methods

### Eligibility

SCCLG-APL is a randomized, multicenter, and non-inferiority trial designed to evaluate whether intravenous ATO can be substituted by oral RIF in the treatment of children (≤ 16 years old) with newly diagnosed APL (Registered at www.clinicaltrials.gov as NCT02200978). The details of the trail design including patients’ selection (Supplemental Figure S1), inclusion and exclusion criteria (Supplemental Table S1), and treatment outcome have been published elsewhere [3]. The present study is a secondary analysis to further compare the impacts of RIF and ATO on DS and coagulation disorder in the pediatric patients. Data from the patients of 8 centers out of the 14 enrolled in SCCLG-APL were retrospectively analyzed. The study was approved by the institutional review board, and informed consent was obtained before treatment in accordance with the Declaration of Helsinki.

### Induction treatment

The details of treatment protocol were published [3]. During induction therapy, patients received ATRA at 25 mg/(m^2^·day) as soon as morphology diagnosis of APL was made. Mitoxantrone (MA) was administrated on day 3 (10 mg/m^2^) or days 2–4 (7 mg/ (m^2^·day) of ATRA treatment for NHR or high-risk (HR) patients (diagnostic WBC > 10 × 10^9^/L) respectively. Once the diagnosis was genetically confirmed (5–6 days later), the patients were randomized to ATO or RIF arm. ATO was administrated at 0.16 mg/(kg·day) (≤ 10 mg/day) intravenously over 12 h daily, while RIF was given at 135 mg/(kg·day) (≤ 30 pills/day) orally three times daily, until complete remission was achieved.

When WBC exceeded 10 × 10^9^/L at the beginning of or during induction treatment, hydroxyurea was administrated at 100 mg/(kg·day). Dexamethasone at 0.3 mg/(kg day) was given if differentiation syndrome or ATRA-associated pseudotumor cerebri was suspected. Transfusions of platelet and fresh-frozen plasma, cryoprecipitate, and/or human fibrinogen (Fbg) were given for the aims of maintaining platelet counts (PLT) greater than 30 × 10^9^/L and Fbg greater than 1.5 g/L, respectively. The use of heparin or low-molecular weight heparins for management of coagulopathy depended on doctors’ clinical judgment.

### Laboratory studies

The dynamic change of WBC and coagulation indexes including PLT, prothrombin time (PT), activated partial thromboplastin time (APTT), d-Dimer, and Fbg were collected on the first day of visit (day 0) and after induction treatment (day 4, 7, 10, 13, 16, 19, 22, 25, and 28 ± 1, respectively). The percentage of promyelocyte in the bone marrow and peripheral blood was determined by microscopic examination by two experienced physicians independently. Minimal residual disease (MRD) was monitored by qRT-PCR for detection of PML-RARα fusion gene.

### Definition of differentiation-related hyperleukocytosis and WBC normalized value

According to our previous study [3], differentiation-related hyperleukocytosis is defined as WBC > 10 × 10^9^/L in NHR arm, and the maximum of WBC increases by over 30% compared with that at diagnosis (day 0) in HR arm during induction treatment. To make the WBC data among patients comparable, the WBC counts have been normalized as the ratio between WBC on day *X* to WBC on day 0. Using this method of normalization, the WBC at day 0 would be 1 for all patients. The calculation formula is: WBC normalized value = $WBC \left(day X\right)\times 100\%/WBC (day 0)$.

### Statistical analysis

Normally distributed variables were expressed as mean ± standard deviation (SD), while median (range) or median (interquartile range) was used to describe skewed variables. To compare the repeated measured data of the RIF and ATO arms, linear mixed model was used. Mann–Whitney *U* test and two independent samples *t* test were used to compare skewed data and normally distributed data respectively. The comparison between dichotomous variables was evaluated by the Chi-square (*χ*^2^) test. ROC curve was used to define the cutoff values. IBM SPSS Statistics 21.0 was used for all statistical analysis.

## Results

### Baseline characteristics of patients

From November 2011 to July 2019, data from 68 consecutive patients were retrospectively analyzed, including 33 in the ATO arm and 35 in the RIF arm. Patient characteristics are showed in Table 1. There were no significant differences in the baseline characteristics between the two arms except for a lower median APTT value in the RIF arm; however, the median values of APTT were normal in both arms.

**Table 1:** Characteristics of patients between ATO and RIF arms

	ATO (*n* = 33)	RIF (*n* = 35)	*p*
Gender (male/female)	24:9	20:15	0.179
Risk stratification (NHR: HR)	22:11	24:11	0.867
Median age, years (range)	7.5 (1.0–13.5)	7.5 (3.2–13.9)	0.602
Hemorrhage on admission, *n* (%)	25 (75.8)	30 (85.7)	0.297
Gastrointestinal	1 (3.0)	1 (2.9)	1.000
Intracranial	2 (6.1)	6 (17.1)	0.298
Important tissue/organ^a^	4 (12.1)	7 (20.0)	0.378
Others^b^	24 (72.7)	28 (80.0)	0.480
Hb, g/L^c^	74 (64–96)	73(62–94)	0.816
WBC, × 10^9^/L^c^	3.95 (1.59–14.84)	5.00 (2.93–12.65)	0.397
< 10 × 10^9^/L, *n*	22	24	0.047
10–50 × 10^9^/L, *n*	7	10	0.601
> 50 × 10^9^/L, *n*	4	1	1.000
PLT, × 10^9^/L^c^	31 (18–61)	22 (14–50)	0.246
d-Dimer, mg/L FEU^c^	15.21 (7.88–20.00)	20.00 (10.29–32.58)	0.138
PT, s^c^	15.4 (13.0–17.1)	15.1 (14.1–16.8)	0.507
APTT, s^c^	37.9 (32.6–42.4)	33.8 (29.7–36.7)	0.030
Fbg, g/L^c^	1.54 (1.19–1.94)	1.40 (1.03–2.04)	0.552
Promyelocytes of blood, %^c^	26.5 (6.3–70.8)	46.0 (10.5–73.5)	0.423
Promyelocytes of bone marrow, %^c^	81.5 (71.0–86.5)	82.3 (70.4–88.5)	0.683
Received hydroxyurea, *n* (%)	13 (39.4)	17 (48.6)	0.446
Received dexamethasone, *n* (%)	22 (66.7)	19 (54.3)	0.297
Time to HCR/HCRp^d^, days (range)	24 (10–41)	25 (14–42)	0.787
Moderate and severe DS, *n* (%)	1 (3.0)	2 (5.7)	0.590

Local reference range of the hematological and coagulation parameters: Hb: 120–140 g/L; WBC: 4.00–10.00 × 10^9^/L; PLT 100–300 × 10^9^/L; d-Dimer, 0.00–0.55 mg/L FEU; PT, 11.0–14.0 s; APTT, 28.0–43.0 s; Fbg, 2.00–4.00 g/L

*NHR* non high risk, *HR* high risk, *Hb* hemoglobin, *WBC* white blood cell count, *PLT* platelet count, *PT* prothrombin time, *APTT* active part thrombin time, *Fbg* fibrinogen

^a^Important tissue/organ includes: fundus hemorrhage, hematuresis, menorrhagia and hemoptysis

^b^Others including petechiae, epistaxis and bleeding gums

^c^Median (interquartile range)

^d^HCR: hematologic complete remission; HCRp: HCR with incomplete platelet recovery [1, 22, 23], one patient in ATO and another in RIF group

### The dynamic change of WBC and the incidence of DS

**Fig. 1:**
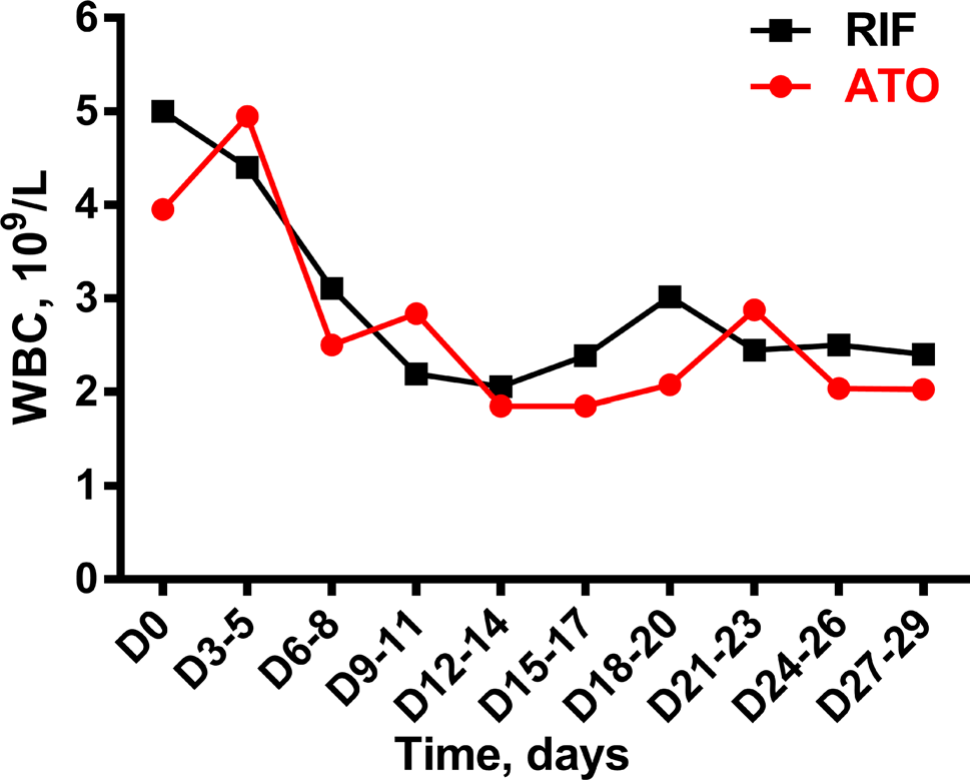
The dynamic changes of median WBC during induction treatment for ATO and RIF arm. The WBC counts at different time points were compared using Mann–Whitney *U* test, all *p* > 0.05. The mixed linear model was used to analyze the overall dynamic trend of the two arms, *p* = 0.539

Combining statistics from NHR and HR patients, the overall dynamic trends of WBC in the ATO and RIF arms during the induction therapy were nearly consistent. Mixed linear model analysis showed that there was no statistical difference between the two arms, *p* = 0.539 (Fig. 1).

**Fig. 2:**
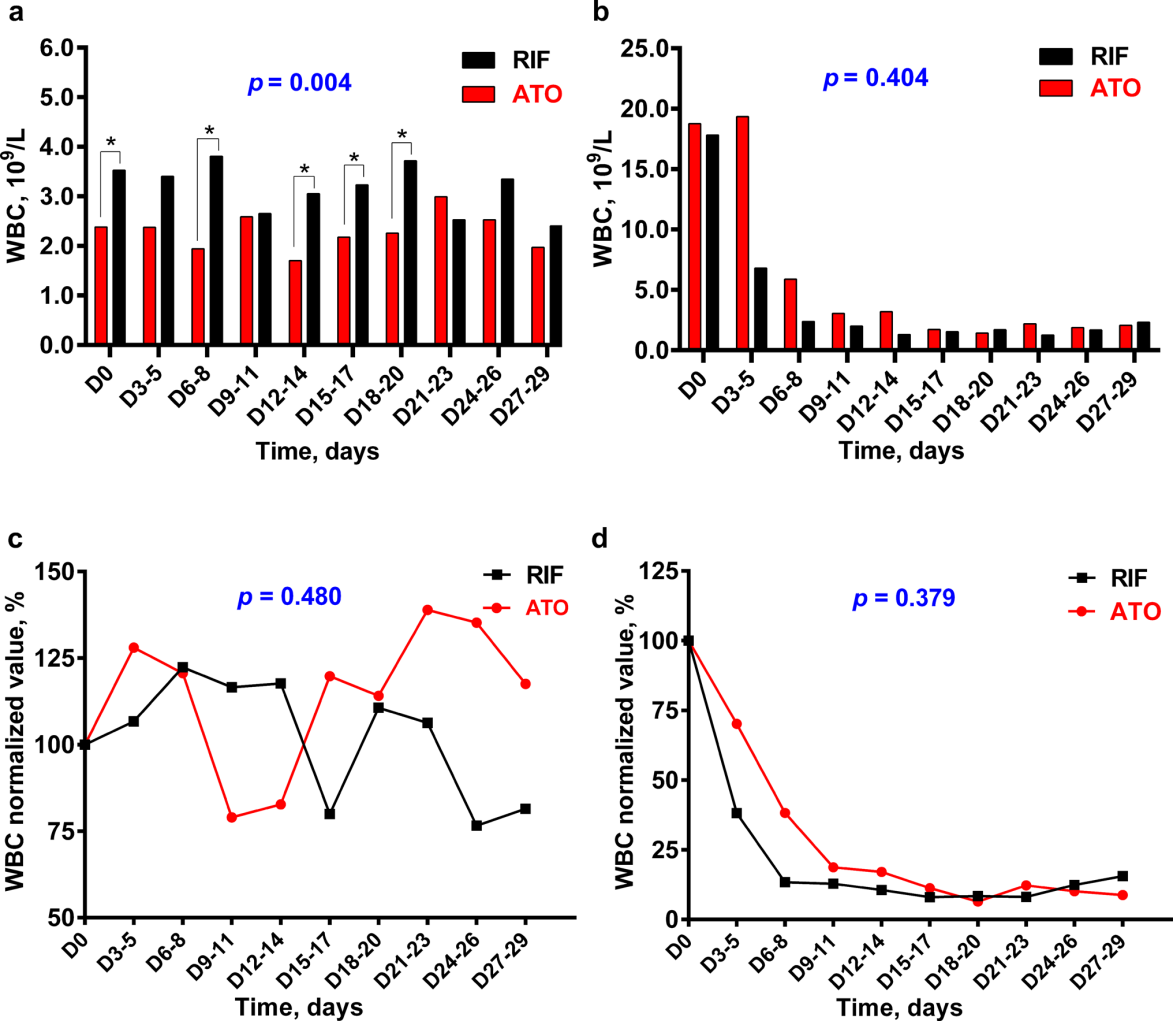
The comparison of dynamic changes of median WBC count and median WBC normalized value between ATO and RIF arm by risk stratification. **a** In NHR arm: There were statistical differences in WBC count between the ATO and RIF arm at five time points. They are D0, days 6–8, days 12–14, days 15–17, and days 18–20 (*p* = 0.047, *p* = 0.034, *p* = 0.035, *p* = 0.044, and *p* = 0.031, separately). Using mixed linear model to analyze the overall dynamic trend of ATO and RIF arms, *p* = 0.004. **b** In HR arm: The overall dynamic trend between ATO and RIF arm was compared by mixed linear model,* p* = 0.404. Using Mann–Whitney *U* test to compare the WBC count at different time points, all *p* value > 0.05 between ATO and RIF arm. **c** In NHR arm: Comparison in the dynamic trend of WBC normalized value between ATO and RIF arms by mixed linear model, *p* = 0.480. Mann–Whitney *U* test was used to compare the WBC normalized value at different time points, *p* > 0.05. **d** In HR arm: The overall dynamic trend of WBC normalized value was compared between ATO and RIF, *p* = 0.379. WBC normalized values at different time point were compared using Mann–Whitney *U* test, all *p* value > 0.05

Next, we separated NHR and HR patients to analyze. As shown in Fig. 2a, NHR patients in the RIF arm had significantly higher WBC at admission and during induction treatment than in the ATO arm. However, when the WBC normalized value was used to evaluate the trends of WBC, there was no significant deference between the two arms (Fig. 2c). The WBC of NHR patients from both arms increased in the first week of induction treatment and then decreased, probably due to the administration of MA on day 3. In HR patients, both the trends of WBC and the normalized values were not statistically different between the ATO and RIF arms (Fig. 2 a and d). The WBC dropped rapidly after induction therapy, which may be related to the use of hydroxyurea at the beginning of induction therapy and the early use of mitoxantrone on the second day of induction therapy.

Moreover, there was no statistical difference in the incidences of moderate and severe DS between the two arms, which was 3.0% and 5.7% in the ATO and RIF arm respectively (*p* = 0.590).

### The risk factors for differentiation-related hyperleukocytosis and DS

Patients with higher WBC counts or higher percentage of promyelocytes in peripheral blood tended to develop differentiation-related hyperleukocytosis (Table 2). As expected, moderate and severe DS occurred in 10.3% and 0% of patients with and without the differentiation-related hyperleukocytosis respectively (*p* = 0.040) (Table 2). Additionally, the incidences of DS were 9.1% and 11.1% in patients with differentiation-related hyperleukocytosis treated with ATO (*n* = 11) and RIF (*n* = 18) respectively (*p* = 0.684) (Table 2). Those patients who developed hyperleukocytosis have higher MRD after induction treatment (*p* = 0.013) (Table 2). Two risk factors were identified for predicting the development of hyperleukocytosis during induction: WBC > 2.61 × 10^9^/L at admission and the percentage of promyelocyte in peripheral blood > 26.5% (Tables 2 and 3). No patient died from DS.

**Table 2:** Characteristics of patients with or without differentiation-related hyperleukocytosis

	With hyperleukocytosis^a^ (*n* = 29)	Without (*n* = 39)	*p*
Gender (male/female)	20:9	24:15	0.526
Median age, years (range)	6.6 (1.0–13.9)	8.2 (1.0–13.5)	0.372
Risk stratification			
HR, *n* (%)	11 (37.9)	11 (28.2)	0.397
NHR, *n* (%)	18 (62.1)	28 (71.8)	
Median WBC at admission, × 10^9^/L^b^	5.18 (3.95–26.58)	2.93 (1.39–10.95)	0.004
Promyelocytes of blood, %^b^	63.5 (27.8–84.0)	14.0 (2.0–55.0)	0.001
Promyelocytes of bone marrow, %^b^	83.0 (77.5–88.0)	80.0 (68.3–86.5)	0.247
Arsenic compound			
ATO, *n* (%)	11 (37.9)	22 (56.4)	0.132
RIF, *n* (%)	18 (62.1)	17 (43.6)	
Duration of arsenical treatment, days	20.0 (5.0–37.0)	20.0 (6.0–38.0)	0.985
MRD^c^, %^b^	0.046 (0.001–0.447)	0.002 (0.000–0.077)	0.013
DS^d^, *n* (%)	3 (10.3)	0	0.040
ATO, *n* (%)	1 (9.1)	0	0.684
RIF, *n* (%)	2 (11.1)	0	

*HR* high risk, *NHR* non high risk

^a^Hyperleukocytosis means differentiation-related hyperleukocytosis

^b^Median value (interquartile range)

^c^MRD: minimal residual disease, PML-RARα/ABL

^d^DS: differentiation syndrome, including moderate and severe forms

**Table 3:** Cutoff values of risk factors to predict differentiation-related hyperleukocytosis

Risk factor	AUC	*p*	Cutoff	Sensitivity	Specificity
WBC at admission, × 10^9^/L	0.704 (0.582–0.827)	0.004	2.61	0.931	0.487
Promyelocytes of blood	0.748 (0.629–0.867)	0.001	26.5%	0.786	0.622

### The recovery of coagulopathy

**Fig. 3:**
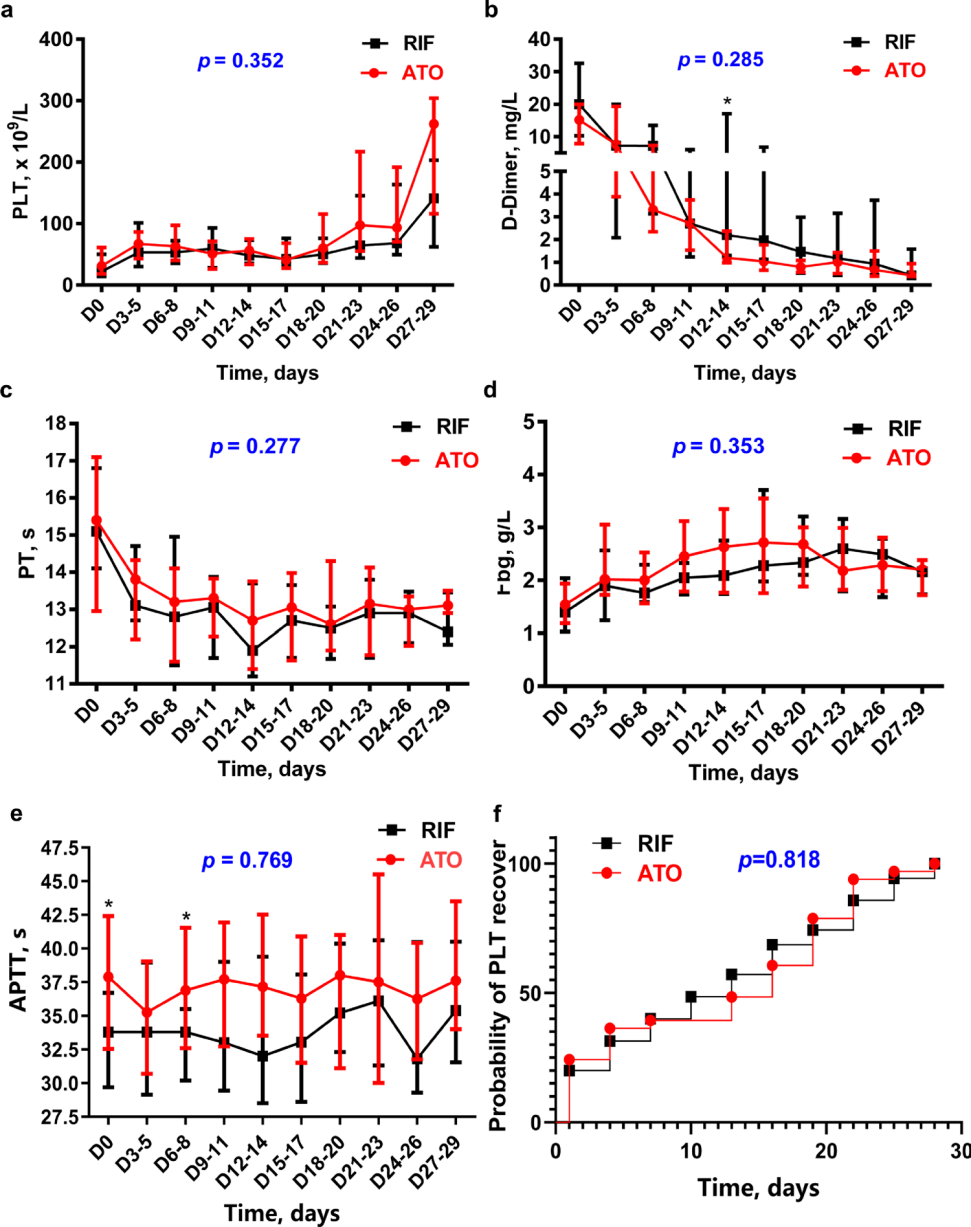
The dynamic trend of coagulation data in ATO and RIF arm. All coagulation data in figure are showed as median with interquartile range. **a**, **c**, **d** Mann–Whitney *U* test was used to compare the difference of PLT, PT, and Fbg between the two arms at each time point, all *p* > 0.05. Using the mixed linear model to compare the recovery trend between ATO and RIF arms, the *p* values of PLT, PT, and Fbg were 0.352, 0.277, and 0.353 respectively. **b** d-Dimer on days 12–14 (*p* = 0.033) showed slower recovery in RIF arm. However, the *p* value of the dynamic trend calculated by mixed linear model was 0.285. **e** Even in normal range, APTT on days 6–8 (*p* = 0.040) recovered slower in ATO arm. **f** Survival analysis comparing the time to platelet recover (PLT > 30 × 10^9^/L) between ATO and RIF

The recovery of coagulopathy was not statistically different between the ATO and RIF arms except for higher values of d-dimer on days 12–14 in the RIF arms (*p* < 0.05) (Fig. 3 and Supplemental Table S2). In general, PT and Fbg recovered on days 3–5. PLT and d-dimer levels needed longer time to recover. At weeks 1, 3, and 4, PLT returned to normal value (100 × 10^9^/L) in 2.9%, 48.5%, and 94.1% of the patients, and d-dimer in 2.9%, 44.1%, and 80.9% of the patients, respectively.

The incidences of bleeding and thrombus events were not statistically different between the ATO and RIF arms. There were 11 (33.3%) patients treated with ATO, and 16 (45.7%) patients treated with RIF had developed coagulopathy during induction (*p* = 0.297). Two cases of peripherally inserted central catheter (PICC) thrombus were observed in the ATO arm. In addition, bleeding events occurred in10 (30.3%) patients treated with ATO and 16 (45.7%) patients treated with RIF, respectively (*p* = 0.191) (Supplemental Table S3). There were 11 (33.3%) and 12 (34.3%) patients requiring heparin treatment in the ATO and RIF arms according to the judgment of clinicians respectively. In addition, the amount of platelets, plasma, and cryoprecipitate used was not statistically different between the two arms (Supplemental Table S4).

## Discussion

We evaluated the trends of WBC counts and compared the values at each time point using the WBC normalized value, as WBC at day 0 varied between patients. Our study found no significant difference in the trends of WBC between the RIF and ATO arms, for both NHR and HR patients. Our data showed that only WBC > 2.61 × 10^9^/L or percentage of promyelocytes in peripheral blood > 26.5% at diagnosis was associated with the development of differentiation-related hyperleukocytosis.

Previous studies reported that adult patients treated with RIF had a significantly higher peak WBC compared to those treated with ATO during induction treatment [8]. However, this was not confirmed in our cohort. The dose of RIF in the previous study (60 mg/(kg day)) was lower than what we used (135 mg/(kg day)). Additionally, the plasma trough concentration of arsenic at steady-state (day 7) was lower in their patients treated with RIF (0.33 μmol/L) compared to those treated with ATO at 0.16 mg/(kg day) (0.75 μmol/L) (*p* = 0.0048) [4]. It has been reported that arsenic at relatively high concentration (0.5 μmol/L or more) mainly induced apoptosis while at low concentration induced differentiation of APL cells [16]. Therefore, it can be explained that a higher peak WBC occurs in patients treated with low dose of RIF at 60 mg/(kg·day) compared to those treated with ATO at 0.16 mg/(kg·day). In our cohort, patients received ATO at 0.16 mg/(kg day) or RIF at 135 mg/(kg day). The plasma trough concentrations of arsenic were similar between the two arms on day 7, which were 0.51 ± 0.16 μmol/L (*n* = 10) and 0.48 ± 0.25 μmol/L (n = 9) (*p* = 0.806) respectively [17], and the trends of WBC were similar. Our data showed that WBC of NHR patients from both arms slightly increased in the first week of induction treatment, even though MA was administrated on day 3. The WBC of HR patients decreased significantly with more intense cytotoxic treatment including hydroxyurea administrated on day 0 and MA on days 2–4. It could be speculated that WBC might obviously increase during induction treatment without cytotoxic therapy.

An important question is raised based on our findings mentioned above. NHR APL in adults has been recently reported that can be successfully treated with a chemotherapy-free combination of ATRA and arsenic compound (RIF or ATO) [14]. However, there is concern that the use of two differentiating agents without chemotherapy may result in an increasing risk of leukocytosis and DS [18]. Previous studies indicated that the incidence of leukocytosis in pediatric patients with NHR APL treated with chemotherapy-free induction therapy was 84–100% and much higher than 35–47% in adult counterpart [10–15]. Recently, two multicenter clinical trials in pediatric APL, CCLG-APL2016 and SCCCG-APL, have been reported [3, 19]. One of the main differences in induction therapy between the two protocols is that the former used chemotherapy-free induction treatment with ATRA and arsenic in NHR patients while the latter used an additional dose of MA besides ATRA and arsenic. The incidence of DS was 6.8 times higher in the CCLG-APL2016 Group (41%) than in the SCCCG-APL group (6%). However, this difference cannot be explained by the difference in the proportion of HR patients between the two groups which is only 1.3 times more in the former than in the latter. The present study showed that WBC > 2.61 × 10^9^/L at diagnosis is a predictor of developing hyperleukocytosis, supporting that pediatric patients with APL are more inclined than adult counterparts to develop hyperleukocytosis during induction treatment. Actually, 2.61 × 10^9^/L is a low WBC value. Our result suggested that leukopenia is a protective factor against developing hyperleukocytosis. Therefore, it strongly suggests that the safety of chemotherapy-free induction proved in adult with NHR APL is questionable in pediatric patients due to much higher incidences of leukocytosis and DS. In fact, even in adult patients, chemotherapy-free induction treatment can cause fatal DS [20].

In addition, our study showed that the recovery of coagulopathy was not statistically different between the ATO and RIF arms except for higher values of d-dimer on days 12–14 in the RIF arms. Our findings support the view that Fbg and PT are early and sensitive indicators of improvement in coagulopathy [21]. A recent study showed that Fbg < 1.0 g/L was independent risk factor for ED in both HR and NHR arms [20]. The recovery of Fbg is therefore essential for the prevention of ED. Our study showed that the recovery of Fbg was the same regardless of whether patients were treated with ATO or RIF, and there was no statistical difference in the incidences of bleeding and thrombus events as well as the consumption of blood components, between the two arms.

There are some limitations of this study. This study is a retrospective analysis based on a multicenter clinical trial. The sample size is relatively small, since only 8 out of the 14 centers which enrolled in SCCLG-APL participated in the present study. Final trial analysis will reveal mature outcome data. Due to the different conditions of each center, it is impossible to conduct more monitoring and comparisons of other indexes related to coagulation.

In conclusion, our study demonstrated the feasibility of replacing ATO at 0.16 mg/(kg day) with RIF at 135 mg/(kg day) in terms of managing two main critical adverse events, DS and hemorrhage, in induction treatment in pediatric APL. Our findings suggest that induction treatment with low-dose chemotherapy, such as anthracyclines, may be important in pediatric APL, as it may decrease the risk of differentiation-related hyperleukocytosis and DS during induction treatment. In pediatric patients, WBC count or percentage of promyelocytes in peripheral blood at diagnosis higher than the cut-off values of 2.61 × 10^9^/L and 26.5%, respectively, can be a risk predictor of developing differentiation-related hyperleukocytosis.

## Supplementary Information

Below is the link to the electronic supplementary material.

Supplementary file1 (DOCX 342 KB)

## Data Availability

The data that support the findings of this study are not openly available to preserve study participant privacy and are available from the corresponding author upon reasonable request.

## References

[1] Iland HJPC, Group ALAL (2015). Use of arsenic trioxide in remission induction and consolidation therapy for acute promyelocytic leukaemia in the Australasian Leukaemia and Lymphoma Group (ALLG) apml4 study: a non-randomised phase 2 trial. Lancet Haematol.

[2] Kutny MA, Alonzo TA, Gerbing RB, Wang YC, Raimondi SC, Hirsch BA (2017). Arsenic trioxide consolidation allows anthracycline dose reduction for pediatric patients with acute promyelocytic leukemia: report from the children's oncology group phase iii historically controlled trial aaml0631. J Clin Oncol.

[3] Yang MH, Wan WQ, Luo JS, Zheng MC, Huang K, Yang LH (2018). Multicenter randomized trial of arsenic trioxide and realgar-indigo naturalis formula in pediatric patients with acute promyelocytic leukemia: interim results of the scclg-apl clinical study. Am J Hematol.

[4] Zhu HH, Wu DP, Jin J, Li JY, Ma J, Wang JX (2013). Oral tetra-arsenic tetra-sulfide formula versus intravenous arsenic trioxide as first-line treatment of acute promyelocytic leukemia: a multicenter randomized controlled trial. J Clin Oncol.

[5] Kayser S, Schlenk RF, Platzbecker U (2018). Management of patients with acute promyelocytic leukemia. Leukemia.

[6] Wang L, Zhou GB, Liu P, Song JH, Liang Y, Yan XJ (2008). Dissection of mechanisms of chinese medicinal formula realgar-indigo naturalis as an effective treatment for promyelocytic leukemia. Proc Natl Acad Sci U S a.

[7] Zhu H, Guo Z, Jia J, Jiang Q, Jiang H, Huang X (2018). The impact of oral arsenic and all-trans-retinoic acid on coagulopathy in acute promyelocytic leukemia. Leuk Res.

[8] Wang F, Jia J, Wang J, Zhao T, Jiang Q, Jiang H (2017). The kinetics of white blood cell and the predictive factors of leukocytosis under oral or intravenous arsenic as the first-line treatment for acute promyelocytic leukemia. Leuk Res.

[9] Kutny MA, Gregory J, Feusner JH (2014). Treatment of paediatric apl: how does the therapeutic approach differ from adults?. Best Pract Res Clin Haematol.

[10] Creutzig U, Dworzak MN, Bochennek K, Faber J, Flotho C, Graf N et al (2017) First experience of the aml-berlin-frankfurt-münster group in pediatric patients with standard-risk acute promyelocytic leukemia treated with arsenic trioxide and all-trans retinoid acid. Pediatr Blood Cancer 64(8):e26461.10.1002/pbc.26461 10.1002/pbc.26461 28111878

[11] Lo-Coco F AGVM, Dell’Adulto GIME, Group GAML, Leukemia SA (2013) Retinoic acid and arsenic trioxide for acute promyelocytic leukemia. N Engl J Med 369:111–121. 10.1056/NEJMoa1300874 10.1056/NEJMoa1300874 23841729

[12] Platzbecker U, Avvisati G, Cicconi L, Thiede C, Paoloni F, Vignetti M (2017). Improved outcomes with retinoic acid and arsenic trioxide compared with retinoic acid and chemotherapy in non–high-risk acute promyelocytic leukemia: final results of the randomized Italian-German apl0406 trial. J Clin Oncol.

[13] Zhang L, Zou Y, Chen Y, Guo Y, Yang W, Chen X (2018). Role of cytarabine in paediatric acute promyelocytic leukemia treated with the combination of all-trans retinoic acid and arsenic trioxide: a randomized controlled trial. BMC Cancer.

[14] Zhu HH, Wu DP, Du X, Zhang X, Liu L, Ma J (2018). Oral arsenic plus retinoic acid versus intravenous arsenic plus retinoic acid for non-high-risk acute promyelocytic leukaemia: a non-inferiority, randomised phase 3 trial. Lancet Oncol.

[15] Zhu HH, Huang XJ (2014). Oral arsenic and retinoic acid for non-high-risk acute promyelocytic leukemia. N Engl J Med.

[16] Chen GQ, Shi XG, Tang W, Xiong SM, Zhu J, Cai X (1997). Use of arsenic trioxide (As2O3) in the treatment of acute promyelocytic leukemia (APL): I. As2O3 exerts dose-dependent dual effects on APL cells. Blood.

[17] Liao LH, Chen YQ, Huang DP, Wang LN, Ye ZL, Yang LH (2022). The comparison of plasma arsenic concentration and urinary arsenic excretion during treatment with realgar-indigo naturalis formula and arsenic trioxide in children with acute promyelocytic leukemia. Cancer Chemother Pharmacol.

[18] Abedin S, Altman JK (2016). Acute promyelocytic leukemia: preventing early complications and late toxicities. Hematol Am Soc Hematol Educ Program.

[19] Zheng H, Jiang H, Hu S, Liao N, Shen D, Tian X et al (2021) Arsenic combined with all-trans retinoic acid for pediatric acute promyelocytic leukemia: Report From the CCLG-APL2016 Protocol Study. J Clin Oncol 39(28):3161–3170. 10.1200/JCO.20.03096 10.1200/JCO.20.03096 PMC8478377 34077242

[20] Wang Y, Hou W, Li H, Tian X, Li J, Hu T (2022). Analysis of risk factors for early death in patients with acute promyelocytic leukaemia treated with arsenic trioxide. Ann Hematol.

[21] Tallman MS, Altman JK (2009). How i treat acute promyelocytic leukemia. Blood.

[22] Ossenkoppele G, Schuurhuis GJ (2016). MRD in AML: does it already guide therapy decision-making?. Hematol Am Soc Hematol Educ Program.

[23] Benjamini O, Dumlao TL, Kantarjian H, O'Brien S, Garcia-Manero G, Faderl S, Jorgensen J, Luthra R, Garris R, Thomas D, Kebriaei P, Champlin R, Jabbour E, Burger J, Cortes J, Ravandi F (2014). Phase II trial of hyper CVAD and dasatinib in patients with relapsed Philadelphia chromosome positive acute lymphoblastic leukemia or blast phase chronic myeloid leukemia. Am J Hematol.

