# Supplementary material for: Differentiation syndrome and coagulation disorder — comparison between treatment with oral and intravenous arsenics in pediatric acute promyelocytic leukemia
Source: Ann Hematol. 2023 May 18;102(7):1713–21. doi: 10.1007/s00277-023-05270-x (PMC10261231; doi:10.1007/s00277-023-05270-x)
Supplement: Supplementary file 1 — Supplementary file1 (DOCX 342 KB) [file 277_2023_5270_MOESM1_ESM.docx]

**Supplementary materials for**

**Differentiation Syndrome and Coagulation Disorder -- Comparison between treatment with Oral and Intravenous Arsenics in Pediatric Acute Promyelocytic Leukemia**

Jie-Si Luo ^1#^，Xiao-Li Zhang ^1#^ , Dan-Ping Huang ^1^, Yi-Qiao Chen ^2^，Wu-Qing Wan ^3^，Hui-Rong Mai ^4^，Hui-Qin Chen ^5^，Hong Wen^6^，Ri-Yang Liu ^7^，Guo-Hua Chen ^8^, Yu Li ^1^, Xue-Qun Luo ^1^，Yan-Lai Tang ^1 *^，Li-Bin Huang ^1 *^

1. Department of Pediatrics, The First Affiliated Hospital, Sun Yat-Sen University, Guangzhou, Guangdong, China.
2. Department of Pediatrics, Fujian Medical University Union Hospital, Fuzhou, Fujian, China.
3. Department of Pediatrics, Second Xiangya Hospital, Changsha, Hunan, China.
4. Department of Hematology and Oncology, Shenzhen Children's Hospital, Shenzhen, China.
5. Department of Pediatrics, Third Affiliated Hospital, Sun Yat-Sen University, Guangzhou, China.
6. Department of Pediatrics, The First Affiliated Hospital of Xiamen University, Xiamen, Fujian, China.
7. Department of Pediatrics, Huizhou Municipal Central Hospital, Huizhou, Guangdong, China.
8. Department of Pediatrics, First People's Hospital of Huizhou, Huizhou, Guangdong, China.

# These authors contributed equally to this study.

*Correspondence: Yan-Lai Tang, [tangylai@mail.sysu.edu.cn](mailto:tangylai@mail.sysu.edu.cn), Li-Bin Huang, [huanglb3@mail.sysu.edu.cn](mailto:huanglb3@mail.sysu.edu.cn). Department of pediatrics, The First Affiliated Hospital, Sun Yat-Sen University, Guangzhou, Guangdong, China.


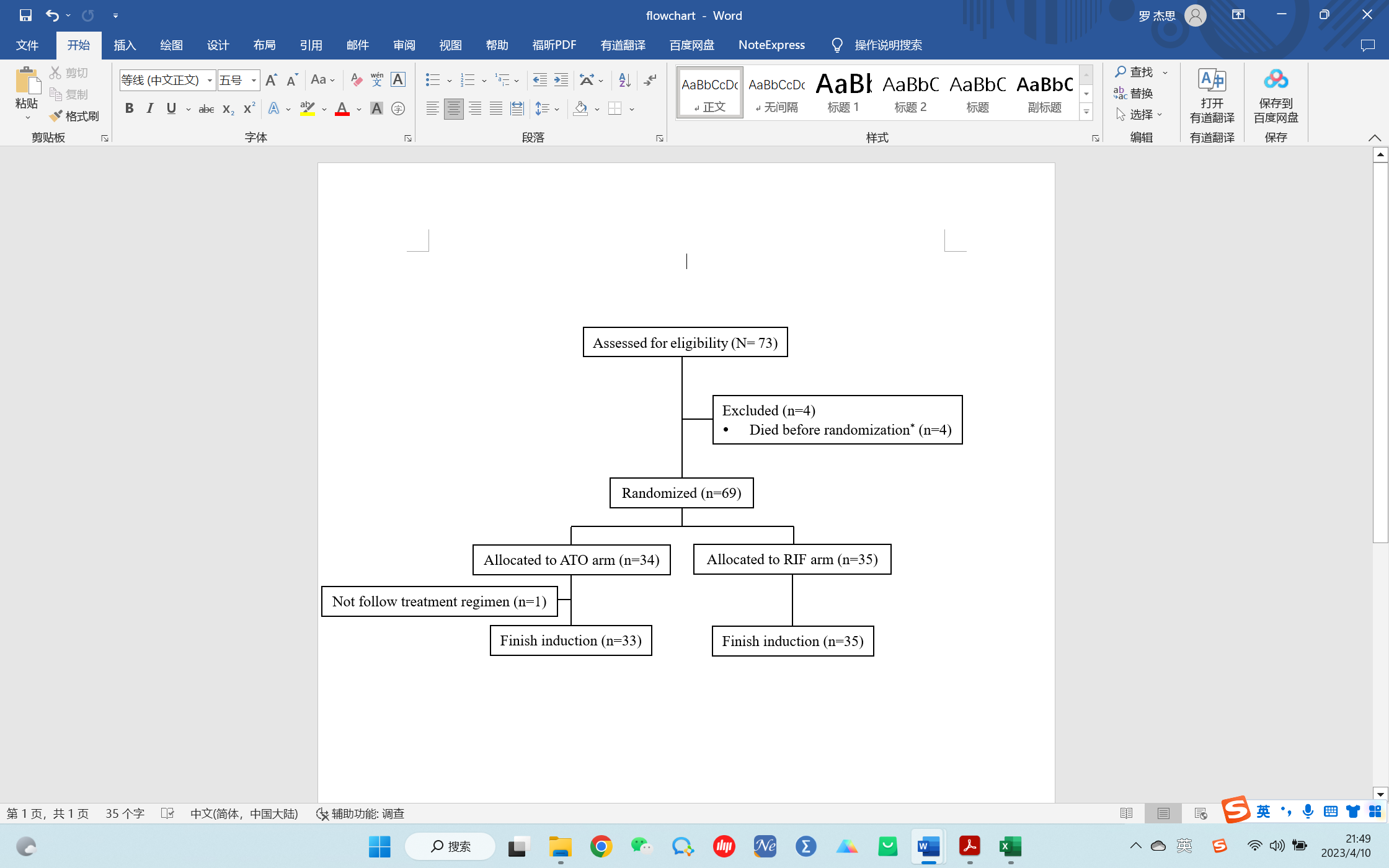


**Supplemental Figure S1 The flowchart of patients’ enrollment.**

**^*^** Two patients cannot afford the treatment and died. Two patients died from DIC and intracranial hemorrhage.

**Supplemental Table S1 Inclusion and exclusion criteria.**

| Inclusion | Exclusion |
| --- | --- |
| - 16 years old or younger with newly diagnosed APL | - Death from any cause |
| - Confirmation of PML-RARα by RT-PCR assay ± FISH^*^ | - Coma, convulsion, paralysis due to   intracranial hemorrhage |
|  | - Cerebral thrombosis |
|  | - Central nervous system leukemia |
|  | - Prolonged QT syndrome |
|  | - Not accept randomization |

^*^ Fluorescence in situ hybridization

**Supplemental Table S2 The cumulative percentage of PLT and blood coagulation indexes recovering to normal.**

|  | PLT | | | |  | Coagulation indexes*^a^* | | | |
| --- | --- | --- | --- | --- | --- | --- | --- | --- | --- |
|  | Total | ATO | RIF | *p^b^* |  | Total | ATO | RIF | *p^b^* |
| At week 1 | 2.9% | 3.0% | 2.9% | 0.838 |  | 2.9% | 0.0% | 5.7% | 0.605 |
| At week 2 | 4.4% | 6.1% | 2.9% |  |  | 13.2% | 12.1% | 14.3% |  |
| At week 3 | 48.5% | 48.5% | 48.6% |  |  | 44.1% | 39.4% | 48.6% |  |
| At week 4 | 94.1% | 97.0% | 91.4% |  |  | 80.9% | 75.8% | 85.7% |  |

*^a^* Include PT, APTT, Fbg and D-Dimer.

*^b^* *p* value was calculated by χ^2^ analysis.

**Supplemental Table S3 Summary of coagulation events during induction treatment.**

|  | Total | ATO | RIF | *p* |
| --- | --- | --- | --- | --- |
| Time of PLT recovery, day | 22(4-43) | 22(7-31) | 22(4-43) | 0.377 |
| Time of correction, day |  |  |  |  |
| PT | 4(4-25) | 4(4-25) | 4(4-25) | 0.727 |
| APTT*^a^* | N/A^*^ | N/A | N/A | N/A |
| D-dimer | 23(10-41) | 23(10-41) | 23(4-39) | 0.508 |
| Fbg | 7(4-28) | 7(4-28) | 10(4-25) | 0.519 |
| Coagulation events during induction, n (%) | 27 (39.7) | 11 (33.3) | 16 (45.7) | 0.297 |
| Intracranial hemorrhage | 4 (5.9) | 1 (3.0) | 3 (8.6) | 0.649 |
| Hematuresis/ hematochezia | 10 (14.7) | 5 (15.2) | 5 (14.3) | 0.920 |
| Other bleeding events^b^ | 17 (25.0) | 7 (21.2) | 10 (28.6) | 0.484 |
| Thrombosis ^c^ | 2 (2.9) | 2 (6.1) | 0 | 0.232 |
| Heparin usage, n (%) | 23 (33.8) | 11 (33.3) | 12 (34.3) | 0.934 |

*^a^* Most of patients had normal APTT at admission, so it is meaningless to compare the correction of APTT.

**^*^** Not applicable.

*^b^* Other bleeding events include epistaxis, gingival bleeding and fundus hemorrhage.

*^c^* Two cases of thrombosis were both Peripherally Inserted Central Catheter (PICC) thrombus.

**Supplemental Table S4 Summary of blood transfusion during induction therapy.**

|  | Total | ATO | RIF | *p* |
| --- | --- | --- | --- | --- |
| PLT transfused, U | 3(0-14) | 2(0-12) | 3(0-14) | 0.306 |
| Last time of PLT transfusion, day | 17(5-36) | 17(5-24) | 17(10-36) | 0.921 |
| Plasma transfused, mL | 0(0-4700) | 0(0-4700) | 0(0-3050) | 0.292 |
| Last time of plasma transfusion, day | 6(1-35) | 5(1-28) | 7(1-35) | 0.799 |
| Cryoprecipitate transfused, U | 0(0-58) | 0(0-14) | 0(0-58) | 0.837 |
| Last time of cryoprecipitate transfusion, day | 6(1-17) | 8(1-16) | 5(1-17) | 0.436 |
